# Prevalence and Clinical Symptoms of Wheat Allergy in Adults and Adolescents in Central Europe

**DOI:** 10.1111/cea.70017

**Published:** 2025-02-19

**Authors:** A. Neyer, S. Dölle‐Bierke, V. Höfer, J. Grünhagen, K. Beyer, M. Worm

**Affiliations:** ^1^ Division of Allergy and Immunology, Department of Dermatology, Venerology and Allergy Charité—Universitätsmedizin Berlin, Corporate Member of Freie Universität Berlin and Humboldt‐Universität zu Berlin Berlin Germany; ^2^ Department of Pediatric Respiratory Medicine, Immunology and Critical Care Medicine Charité—Universitätsmedizin Berlin, Corporate Member of Freie Universität Berlin and Humboldt‐Universität zu Berlin Berlin Germany; ^3^ Partner Site Berlin German Center for Child and Adolescent Health (DZKJ) Berlin Germany

**Keywords:** anaphylaxis, food allergy, food sensitivity, prevalence, self‐reported, wheat

## Abstract

**Background:**

Wheat is a well‐known elicitor of food allergy, but epidemiological data are limited. The aim of this study was to investigate the prevalence of wheat allergy in an unselected population of adults and adolescents and to characterise the clinical features of this cohort, as well as those of patients who experienced wheat‐induced anaphylaxis.

**Methods:**

A population‐based cross‐sectional study was conducted. Fifteen thousand individuals aged 12–80 years were randomly selected, and a standardised questionnaire was applied. If symptoms after wheat consumption were reported, telephone interviews were conducted. In the case of suspected type 1 wheat allergy, a skin prick test, specific immunoglobulin E (sIgE) and oral food challenge (OFC) were performed. The prevalence of self‐reported wheat sensitivity and wheat allergy was determined after data extrapolation. For the assessment of severe wheat allergy‐associated symptoms, a cohort from the European Anaphylaxis Registry was analysed.

**Results:**

The questionnaire was answered by 1770 individuals, of whom 13.1% reported symptoms due to the consumption of foods containing wheat. Following telephone interviews (*n* = 105) and clinical diagnostics (*n* = 22), type 1 sensitisation to wheat was confirmed in 8 individuals, and 2 subjects were finally diagnosed with an IgE‐mediated wheat allergy. After extrapolation, the prevalence of confirmed wheat allergy in the German population reached 0.25% [95% CI 0.08–0.9]. Self‐reported wheat sensitivity was predominantly seen in females (71%), with local gastrointestinal and non‐specific symptoms. This contrasted with wheat‐induced anaphylaxis, where less than half occurred in females, and symptoms were mainly skin, cardiovascular or respiratory.

**Conclusion:**

In a population with widespread wheat consumption, self‐reported wheat sensitivity was common in adults and adolescents, but confirmed wheat allergy was rare. The distinct symptom profiles allow physicians to easily differentiate these entities. Dissemination of our findings may help to improve knowledge of the low prevalence of wheat allergy and may support the reduction of unnecessary dietary restrictions.

## Graphical abstract

Self‐reported wheat sensitivity is frequent in adults and adolescents in the general population and higher in females. Individuals with self‐reported wheat sensitivity report a distinct symptom pattern (local gastrointestinal and unspecific symptoms). In contrast, our data confirm that IgE‐dependent wheat allergy is rare in adults and adolescents in central Europe.

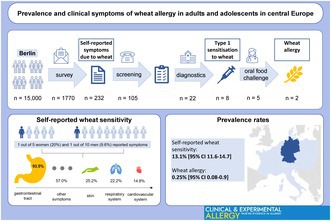

**Summary**

- Self‐reported wheat sensitivity is frequent in adults and adolescents and is higher in females.
- IgE‐dependent wheat allergy in adults and adolescents is rare in central Europe.
- Self‐reported wheat sensitivity and wheat‐induced anaphylaxis are characterised by distinct clinical symptoms.

## 1 Introduction

Wheat ( *Triticum aestivum* ) is a staple food in the diet worldwide, and its predicted global utilisation reaches 794 million tonnes in 2023/24 [1, 2, 3]. Nevertheless, wheat and its components are well‐known food allergens [4]. The clinical manifestations of immunoglobulin (Ig)E‐dependent wheat allergy may present as respiratory allergy (Baker's asthma), food allergy, wheat‐dependent exercise‐induced anaphylaxis (WDEIA) or contact urticaria [4]. Previous data from the European Anaphylaxis Registry indicate that wheat is a common trigger of food‐induced anaphylaxis [5, 6, 7].

Unlike many other foods (e.g. apples, cow's milk), wheat is widely consumed in a processed manner, which can lead to a modification of the allergenic proteins [8]. Gluten is a major allergen in wheat allergy and mainly contains gliadin and glutenin [9]. Along with other proteins such as albumin and globulin, these protein fractions are characterised by different solubility [9]. Currently, 28 different allergens have been described for *T. aestivum* according to the list of the World Health Organisation/International Union of Immunological Societies Allergen Nomenclature Sub‐Committe [10]. Omega‐5 gliadin (Tri a 19) has been reported as the major allergen in patients suffering from WDEIA [11, 12, 13, 14]. Further allergens of wheat are alpha‐, beta‐ and gamma‐gliadins, low molecular weight (LMW) glutenins, high molecular weight (HMW) glutenins and alpha‐amylase inhibitors [15, 16, 17, 18, 19]. Wheat also contains a lipid transfer protein (LTP Tri a 14), which has been reported to be clinically relevant in baker's asthma [20] but also in food‐induced wheat allergy [17, 21, 22].

The diagnostic workup of wheat allergy includes a detailed history, the determination of specific immunoglobulin E (sIgE) and/or a skin prick test [23]. As these tests can only determine a sensitisation, the oral food challenge (OFC) is the diagnostic gold standard to confirm a clinically relevant wheat allergy [4, 23].

Symptoms of IgE‐mediated wheat allergy may manifest in various organ systems and include urticaria, angioedema, rhinitis, erythema, wheezing, asthma, vomiting and abdominal pain [15, 24, 25]. Previous studies have also shown that severe allergic reactions due to wheat in adults are more frequently associated with the onset of cardiovascular symptoms [7, 26]. By contrast, non‐celiac gluten sensitivity (NCGS) is classified as a non‐autoimmune‐non‐allergic reaction, which is an exclusionary diagnosis and a controversially discussed clinical entity [4, 27, 28]. NCGS has been reported to be associated with intestinal (e.g. diarrhoea, abdominal pain) and many extraintestinal manifestations (e.g. muscle cramps, bone or joint pain, tiredness, headache) [28, 29, 30].

Data on the prevalence of wheat allergy is limited, in particular for adults. Previous data suggested a lifetime prevalence of parent‐reported doctor‐diagnosed wheat allergy in children in Germany of 0.4% [31]. Data from Finland showed a prevalence of self‐reported physician‐confirmed wheat allergy in children aged 1–4 years of 2.6% [32]. A multicentre cross‐sectional European study suggested a prevalence of probable wheat allergy (based on symptoms and sIgE) for adults of up to 0.37% [33].

In this study, we performed a population‐based study on the prevalence of self‐reported wheat sensitivity and confirmed wheat allergy in Germany and performed a comparative analysis with a cohort of patients who experienced wheat anaphylaxis.

## 2 Methods

### 2.1 Study Design and Study Population

A population‐based, cross‐sectional study to determine the prevalence of wheat allergy in Germany (PAN‐WA) was conducted from October 2021 to June 2022 at the Division of Allergy and Immunology in the Department of Dermatology, Venereology, and Allergology, Charité—Universitätsmedizin Berlin, Germany. For data collection, a random sample of the Berlin population (*n* = 15,000) was surveyed. The sample size calculation was based on the expected prevalence of wheat allergy of 1% with a two‐sided 95% confidence interval smaller than [0.5–1.5] and resulted in a required sample size of 1500 individuals. With an estimated response rate of 10%, this led to a target study population of 15,000 persons. On two survey dates (11/2021 and 02/2022), 15,000 individuals randomly selected from the Berlin Population Registry were contacted by mail and asked to anonymously complete an electronic questionnaire. Subjects who reported symptoms after wheat consumption were asked to provide their informed consent to be contacted by the study team. Individuals between 12 and 80 years of age registered in Berlin were included, with a balanced gender distribution between men and women (50:50). The implementation of all study steps was approved by the ethics committee (EA2/136/21) and the data protection department of Charité—Universitätsmedizin Berlin.

### 2.2 Questionnaire and Telephone Interviews

**FIGURE 1:**
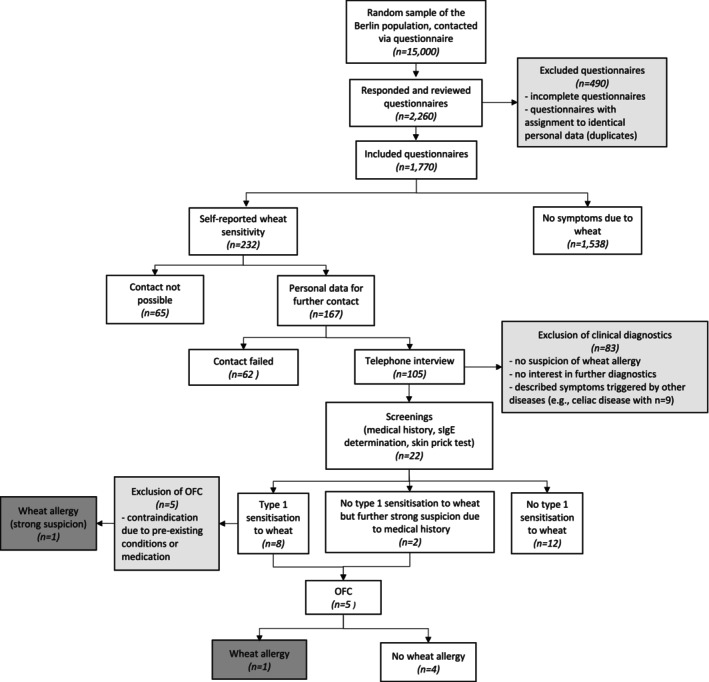
Flow chart of the PAN‐WA study sample. OFC, oral food challenge; sIgE, specific Immunoglobulin E.

A standardised anonymous questionnaire was developed to survey the sample, with a version for adults and a version for legal guardians of adolescents aged 12–17 years (Document S1). After a qualitative pre‐test of the reliability and validity of the questionnaire, it was implemented in a web application system for electronic completion. Demographic data, symptoms caused by the consumption of foods containing wheat and their relation to cofactors were acquired. The questionnaire also addressed (atopic) pre‐existing conditions and complaints caused by other foods. In the next step, telephone interviews were conducted by trained personnel with the individuals who reported symptoms after the consumption of wheat‐containing foods in the questionnaire. These served to clarify whether the complaints reported in the questionnaire could be linked to a wheat allergy. Criteria indicating the possible presence of a wheat allergy were, for example, the reproducibility of the symptoms upon repeated consumption of wheat, the temporal association between ingestion and the onset of symptoms, no previously diagnosed celiac disease as the trigger of the symptoms described, and the occurrence of objective symptoms involving at least one of the four organ systems queried. In the case of a suspected wheat allergy, further diagnostics were performed in our division. Figure 1 shows a consort diagram of the study.

### 2.3 Clinical Diagnostics

**FIGURE 2:**
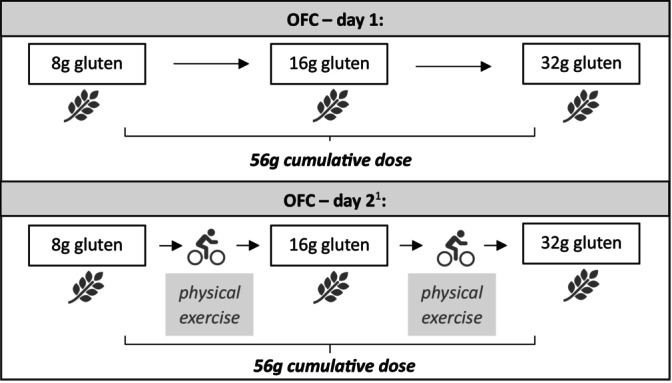
OFC procedure on day 1 and 2. ^1^Day 2 of the OFC was only carried out if there was no allergic reaction to wheat on day 1. OFC, oral food challenge.

The clinical diagnostics included a history and blood sampling for the determination of the total IgE value, wheat sIgE (wheat extract, gluten, gliadin, Tri a 14, Tri a 19) and the detection of tryptase. A skin prick test was conducted using wheat flour and wheat gluten (prick‐to‐prick) and pollen allergen extracts (birch, mugwort, timothy grass, house dust mite and cat, all from ALK‐Abelló, Hamburg, Germany), besides histamine as a positive control (10 mg/mL from ALK‐Abelló) and 0.9% sodium chloride as a negative control. Type 1 sensitisation to wheat was defined as elevated sIgE levels ≥ 0.35 IU/mL and/or a positive SPT with a wheal to wheat flour or wheat gluten ≥ 3 mm after 15 min. OFC according to protocols published previously [34, 35] was performed if type 1 sensitisation was proven (Figure 2). Exclusion criteria for OFC were the presence of severe disease (e.g., uncontrolled bronchial asthma), treatment with beta‐blockers, ACE inhibitors, or biologicals, use of antihistamines 3–5 days before OFC, pregnancy or lactation, acute infection or systemic immunosuppression.

### 2.4 Data From the Anaphylaxis Registry (Network for Online Registration of Anaphylaxis, NORA e.V.)

Data from the Anaphylaxis Registry were extracted as previously described [7, 26] for comparative analysis of the clinical symptoms of wheat allergy. Cases of wheat‐induced anaphylaxis in adults reported between 2007 and 2023 from Germany, Poland, Bulgaria, Italy, Spain, Austria, Switzerland, France and Romania were selected.

### 2.5 Statistical Analysis

Statistical analysis was performed using IBM SPSS Statistics 27. Descriptive analysis involved the calculation of mean values with a standard deviation or the median with the interquartile range (IQR). Categorical variables were analysed in terms of absolute and relative frequencies. A correlation test between two categorical variables was performed using the Pearson's Chi‐squared test. Weighting of the data was performed to calculate the point prevalence of wheat allergy and the associated extrapolation to Berlin and Germany. Weighting factors (age and gender distribution) within the Berlin and German populations were generated and are shown in Table S1 [36, 37]. The point prevalence of clinically confirmed wheat allergy in Berlin and Germany is given with a confidence interval of 95% and was determined by means of a Bayesian credibility interval.

## 3 Results

### 3.1 Descriptive Data From the Population‐Based Sample

#### 3.1.1 Baseline Characteristics

The questionnaire assessing the presence of wheat‐dependent symptoms was answered by 1770 individuals (response rate of 12%). Of the study participants, 55% were female, and the median age in this cohort was 46 years (IQR 29–62). The proportion of adolescents aged 12–17 in the sample reached 13%. The frequency of individuals with a university degree answering the questionnaire was 36.6%.

#### 3.1.2 Self‐Reported Symptoms Due to Wheat

Symptoms after the consumption of wheat‐containing foods were reported by 232 individuals (13.1%; median age 47, IQR 32–59). Of these, only 7.8% were adolescents. Up to 20% of all females (162/811) indicated a self‐reported wheat sensitivity, but only 9.6% of all men (70/726). Of the individuals with self‐reported wheat sensitivity, 37.7% had a university degree. Overall, 57% of respondents indicated that they had experienced their symptoms for 5 years or longer. Regarding the frequency of symptoms, 77.7% reported complaints at least once a week, and 74.7% developed their symptoms 2 h after consumption of wheat‐containing foods. Considering affected organ systems (skin, respiratory system, gastrointestinal tract, cardiovascular system), 61.3% of the individuals reported symptoms from one organ system, 23.9% had symptoms from two organ systems and in only 6% of cases were three organ systems affected.

The vast majority of reported symptoms (93.5%) were related to the gastrointestinal tract, and 57% of the individuals reported unspecific symptoms such as heat, dizziness or fatigue. Furthermore, 25.2% reported skin and 22.2% respiratory symptoms (Table S2). The most frequently reported symptoms were bloating, abdominal pain, diarrhoea, decreased sense of well‐being and fatigue (Table S2).

#### 3.1.3 Cofactors and Pre‐Existing Conditions

The most frequently reported cofactors were psychological burden (40.6%) and alcohol consumption (12.2%), while only 5.7% of subjects suspected physical exercise as a cofactor. We observed that 37.5% of individuals with self‐reported wheat sensitivity had pre‐existing atopic conditions, compared to 28.5% of those who did not experience symptoms related to wheat. Among individuals with self‐reported wheat sensitivity, the following atopic diseases were reported: allergic rhinitis at 27.2%, bronchial asthma at 13.8%, atopic dermatitis at 10.4% and urticaria at 2.6%. In terms of other pre‐existing conditions, we observed that 17/232 participants with wheat‐induced symptoms stated that they suffered from celiac disease, while 36/232 reported that they suffered from irritable bowel syndrome.

#### 3.1.4 Previous Treatment and Diagnostics

Of individuals with self‐reported wheat sensitivity, 86.6% have changed their diet due to symptoms (55% reduction of foods containing wheat; 31.6% strict avoidance of wheat or gluten). Of these, 73.2% experienced an improvement in symptoms from dietary changes. Additionally, 47.8% sought advice from a physician (e.g., a family doctor (68.5%), an allergologist (19.8%) or a nutritionist (16.2%)).

### 3.2 Type 1 Sensitisation and Diagnosis of Wheat Allergy

Type 1 sensitisation to wheat was detected in 8/22 individuals with self‐reported wheat sensitivity, determined by skin prick test and/or sIgE (Figure 1; Table 1). OFC was carried out in 5 subjects, confirming IgE‐mediated wheat allergy in one of them. Another subject was confirmed with wheat allergy based on a convincing medical history and the detection of sensitisation (skin prick test and sIgE) only due to a contraindication for OFC. Finally, we diagnosed 2 subjects with clinically confirmed wheat allergy.

**TABLE 1:** Results of allergy testing of *n* = 22 individuals with self‐reported wheat sensitivity using a skin prick test and blood sampling to detect type 1 sensitisation to wheat.

Subjects	Gender	Age	Type 1 sensitisation to wheat	Total IgE (kU/L)	sIgE wheat extract (kU/L)	sIgE total gluten (kU/L)	sIgE total gliadin (kU/L)	sIgE tri a 19 (kU/L)	sIgE tri a 14 (kU/L)	Tryptase (μg/L)	Histamine (positive control)	0.9% sodium chloride (negative control)	Wheat flour	Wheat gluten	Birch	Mugwort	Timothy gras	House dust mite	Cat
1	M	35	No	285	0.19	< 0.10	0.01	< 0.10	< 0.10	4.15	8	2	2.5	2	8.5	6.5	8	7.5	5
2	M	34	No	184	< 0.10	< 0.10	0	< 0.10	< 0.10	4.13	6	0	0	0	8.5	5.5	5.5	5	10
3	W	47	Yes	390	0.68	0.14	< 0.10	< 0.10	< 0.10	11.2	6	0	2	0	6	4	10.5	0	5
4	M	47	No	30.7	< 0.10	< 0.10	< 0.10	< 0.10	< 0.10	4.93	8.5	0	0	0	0	0	0	0	0
5	W	51	No	8.48	< 0.10	< 0.10	< 0.10	< 0.10	< 0.10	2.46	8	0	0	0	0	0	0	0	0
6	W	57	Yes	38.8	< 0.10	< 0.10	< 0.10	< 0.10	< 0.10	5.13	7.5	0	0	3	3	0	2	2	0
7	M	30	No	270	0.27	0.14	< 0.10	< 0.10	< 0.10	6.01	7	0	2	2	3	2	4.5	0	0
8	W	28	No	366	< 0.10	< 0.10	< 0.10	< 0.10	< 0.10	1.53	8.5	0	0	0	0	0	3	3	3.5
9	M	33	No	14.7	< 0.10	< 0.10	< 0.10	< 0.10	< 0.10	4.2	8.5	0	0	0	0	0	0	0	0
10	W	45	Yes	22.4	< 0.10	< 0.10	< 0.10	< 0.10	< 0.10	4.4	9	0	0	3	3.5	0	0	2	0
11	M	77	Yes	774	6	8.19	0.87	0.71	1.96	5.82	8	0	5.5	3.5	6	0	5	0	6
12	W	53	No	58.5	< 0.10	< 0.10	< 0.10	< 0.10	< 0.10	5.29	8	0	2	0	3.5	0	0	0	5
13	M	34	Yes	88	< 0.10	< 0.10	< 0.10	< 0.10	< 0.10	4.17	9	0	4	3	0	0	0	5	5
14	W	55	Yes	51.4	< 0.10	< 0.10	< 0.10	< 0.10	< 0.10	6	8	0	5	4	0	3	0	0	3
15	M	34	No	127	< 0.10	< 0.10	< 0.10	< 0.10	< 0.10	3.74	9	0	0	2	2	5	2	5	3.5
16	W	57	No	24.8	< 0.10	< 0.10	< 0.10	< 0.10	< 0.10	3.34	8.5	0	0	2	2	0	0	0	2
17	M	63	No	4.12	< 0.10	< 0.10	< 0.10	< 0.10	< 0.10	7.13	7.5	0	0	0	0	0	0	0	0
18	W	31	No	3.25	< 0.10	< 0.10	< 0.10	< 0.10	< 0.10	1.72	7	0	0	0	0	0	0	0	0
19	W	53	Yes	114	< 0.10	< 0.10	< 0.10	< 0.10	< 0.10	18	7.5	0	0	4	0	0	3	0	0
20	M	20	Yes	719	0.75	< 0.10	< 0.10	< 0.10	< 0.10	2.63	8.5	0	7.5	8	9	5	9	10	3.5
21	W	68	No	< 2.0	< 0.10	< 0.10	< 0.10	< 0.10	< 0.10	7.5	9	0	0	0	0	0	0	0	0
22	W	53	No	164	< 0.10	< 0.10	< 0.10	< 0.10	< 0.10	12.9	10	0	0	0	10	0	9	6.5	0

Abbreviations: IgE, Immunoglobulin E; sIgE, specific Immunoglobulin E.

### 3.3 Prevalence of Self‐Reported Wheat Sensitivity and Clinically Confirmed Wheat Allergy

To determine the prevalence of self‐reported wheat sensitivity and clinically confirmed wheat allergy, the data was weighted for extrapolation (Table S1). The point prevalence of self‐reported wheat sensitivity reached 13.3% [95% CI 11.7–14.9] for Berlin and 13.1% [95% CI 11.6–14.7] for Germany.

Within the Berlin population, there was a difference (*p* < 0.001) between the point prevalence of self‐reported wheat sensitivity in men (9.0% [95% CI 7.09–10.9]) and women (17.5% [95% CI 15–20]). The point prevalence for adults was 13.5% [95% CI 11.9–15.2], and for adolescents, it was 9.5% [95% CI 4.2–14.8] (*p* = 0.214).

Similarly, after the extrapolation for Germany, women were more likely (*p* < 0.001) to report wheat sensitivity than men (17.5% [95% CI 15–20] versus 8.8% [95% CI 6.9–10.7]). Accordingly, the point prevalence for adults reached 13.4% [95% CI 11.8–15], while only 9.6% [95% CI 4.4–14.6] of all adolescents were affected (*p* = 0.225).

**FIGURE 3:**
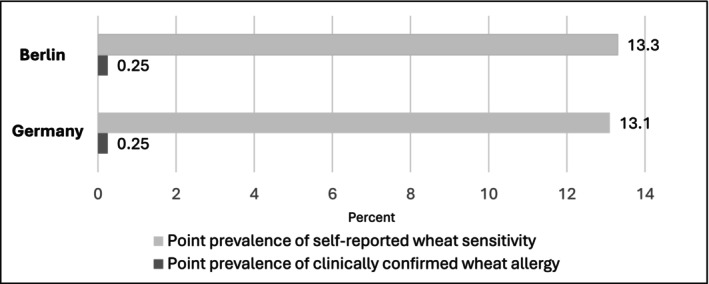
Point prevalence of self‐reported wheat sensitivity and clinically confirmed wheat allergy in adults and adolescents.

Based on the clinical data, an extrapolation for confirmed wheat allergy was possible. The calculated point prevalence of confirmed wheat allergy reached 0.25% for the Berlin population [95% CI 0.08–0.87] and 0.25% [95% CI 0.08–0.9] for the German population. The differences between the point prevalences of self‐reported wheat sensitivity and clinically confirmed wheat allergy are depicted in Figure 3.

### 3.4 Self‐Reported Symptoms of Wheat Sensitivity Versus Wheat Anaphylaxis in Adults

In the European Anaphylaxis Registry, 284 cases of wheat‐dependent anaphylactic reactions in adults were registered between 2007 and 2023 (181 coming from Germany). These were analysed in a comparative manner to the 214 adult cases of self‐reported wheat sensitivity from our cohort. The gender ratio was balanced in the anaphylaxis cohort (Europe: 40.8% female; Germany: 48.6% female), whereas the majority of individuals in our wheat sensitivity cohort were female (71%). Anaphylactic patients developed mainly skin or cardiovascular symptoms, followed by respiratory symptoms (e.g., urticaria, hypotension, dyspnoea). Individuals from the wheat sensitivity cohort experienced mainly gastrointestinal and other symptoms (e.g., flatulence, abdominal pain, diarrhoea). While in self‐reported wheat sensitivity, mostly one organ system was affected, the anaphylactic reactions to wheat from the registry included patients who experienced the involvement of mostly two or three organ systems. The most frequent cofactor for subjects with self‐reported wheat sensitivity was psychological burden (39.9%), followed by alcohol consumption and menstruation (12.1% each). Physical exercise (Europe: 81.3%; Germany: 80.7%) and medication intake (Europe: 34.7%; Germany: 40.1%) were the predominantly reported cofactors in anaphylactic reactions. Table 2 shows the detailed data for the comparison of the PAN‐WA cohort with the NORA cohort.

**TABLE 2:** Symptom profile of the PAN‐WA cohort with self‐reported wheat sensitivity compared with the NORA cohort in Europe and Germany with an anaphylactic reaction to wheat.

	PAN‐WA (*n* = 214)	NORA—Europe (*n* = 103)	NORA—Germany (*n* = 181)
Median age (years)	50.5 (IQR 34–61)	42 (IQR 32–56)	39 (IQR 30–52)
Gender	71% female	40.8% female	48.6% female
Affected organ systems (%)			
No organ system (only subjective symptoms)	1.9	—	—
One organ system	61.2	1.9	0.6
Two organ systems	24.3	46.6	40.9
Three organ systems	7.0	42.7	41.4
Four organ systems	5.6	8.7	17.1
Symptom profile (%)			
Skin	24.8	96.1	98.3
Respiratory system	21.5	53.5	54.0
Gastrointestinal tract	92.5	32.4	41.5
Cardiovascular system	14.5	80.2	86.0
Other symptoms	55.6	21.1	33.9
Most common symptoms			
1	Flatulence	Urticaria	Dyspnoea
2	Abdominal pain	Dyspnoea	Urticaria
3	Diarrhoea	Angioedema	Pruritus
4	Abdominal cramps	Hypotension	Hypotension
5	Constipation	Loss of consciousness	Angioedema
Most frequent cofactors (%)			
Psychological burden	39.9	5.8	19.9
Alcohol consumption	12.1	21.9	21.9
Menstruation	12.1	14.3	7.4
Physical exercise	5.1	81.3	80.7
Medication intake	4.2	34.7	40.1

## 4 Discussion

Our data confirm that wheat allergy is not frequent in adults and adolescents in the German population, reaching a prevalence of 0.25% [95% CI 0.08%–0.9%] in this study. Given the high population number in Germany, we suppose that the prevalence of wheat allergy in central Europe is also not frequent [38]. Comparing our extrapolated data from clinically confirmed wheat allergy with data from other studies, similarly low prevalences of less than 1% were reported. A meta‐analysis on the prevalence of wheat allergy showed a prevalence of OFC‐confirmed wheat allergy of 0.12% [95% CI 0.63%–17.77%] in European regions [39]. Another meta‐analysis showed that the prevalence of wheat allergy confirmed by OFC was similar at 0.1% [95% CI 0.01–0.2%] in Europe [40]. In addition, data from the cross‐sectional study in EuroPrevall show prevalence rates of probable wheat allergy (based on typical clinical symptoms and sIgE) in children of up to 0.21% [41] and of up to 0.37% in adults [33]. Looking directly at Germany, a lifetime prevalence for parent‐reported doctor‐diagnosed wheat allergy in children from the EuroPrevall birth cohort is estimated at 0.4% [31].

Previously reported cross‐sectional studies from Europe indicate a range of prevalence for self‐reported wheat allergy in children between 0.2% and 1.5% [32, 42, 43]. In addition, 2.6% of parents in Finland stated that their child had a physician‐confirmed wheat allergy [32]. Two studies from Germany provided information on sensitisation to wheat determined by sIgE or skin prick test, reporting a prevalence of 9.9% for children [44] and 4.7% for all age groups [45].

The prevalence rates of wheat allergy show a wide range worldwide. A study from Japan reported a prevalence of probable wheat allergy in adults of 0.21% [46] whereas a study from South India reported a prevalence of 0.02% [47]. Eating behaviours like differences in the amount and/or way of processing of wheat‐containing foods, besides the socioeconomic conditions and genetic background of a population, have an impact on the overall prevalence of wheat allergy [48].

Overall, our study indicates that wheat is a rather rare food allergen in adults and adolescents. In contrast, other food allergens, for example, peanut [49, 50] and tree nut [49] allergies, have been increasingly reported worldwide in recent decades [51, 52]. Possible reasons might be differences in exposure but also the allergenicity of the proteins, which may be altered by thermal processing, for example, cooking, resulting in less allergenicity [53, 54, 55] or roasting with increased allergenicity [54, 56, 57]. The allergenicity of wheat has been shown to be indirectly altered through yeast‐ and bacteria‐dependent fermentation processes, but also directly through the thermal processing of wheat proteins [8]. On the other hand, wheat is a staple food and has been consumed for thousands of years [58], resulting rarely in clinically relevant manifestations with the exception of celiac disease, reaching a prevalence of around 1%–2% worldwide [59, 60, 61, 62, 63].

Previous studies have shown that the skin prick test and the detection of sIgE to wheat extract have low specificity and low positive predictive values for the diagnosis of wheat allergy [24, 64]. One reason is the cross‐reactivity between wheat and grass pollen [24, 64]. In contrast, it has been demonstrated that a skin prick test with gluten and/or the determination of omega‐5‐gliadin‐sIgE is associated with high sensitivity and specificity, particularly for the diagnosis of WDEIA [34, 65]. Therefore, their application (sIgE measurement and skin prick test) as the first step of a diagnostic workup should be considered. Whenever the diagnosis is unclear, an OFC should be performed as the gold standard [66]. However, due to the presence of possible concomitant diseases or other exclusion criteria, adult individuals may not be challenged; for example, in this study, in five subjects the OFC could not be performed.

Our data also indicate a high prevalence of self‐reported wheat sensitivity, reaching 13.1% [95% CI 11.6–14.7] in the German population. A study from the UK on self‐reported gluten sensitivity showed similar data, with a prevalence of self‐reported gluten sensitivity of 13% in individuals over 16 years old, based on a population‐based questionnaire [67]. Another cross‐sectional study from Argentina presented lower values for the prevalence of self‐reported gluten sensitivity of 7.61% (6.2–9.2) in adults [68]. In both studies, more women indicated self‐reported gluten sensitivity, which is consistent with the predominance of the female gender of self‐reported wheat sensitivity in our study [67, 68]. These gender differences of self‐reported food‐related complaints were also observed in other previous studies [69, 70]. Marklund et al. reported in their study on food hypersensitivity in 13–21‐year‐olds that women had more frequent symptoms than men [69]. Another study revealed a predominance of the female gender in self‐reported food allergies/food intolerances [70]. In addition to the increased risk of women reporting food‐related symptoms, they also appear to be more likely to react positively to OFCs [71].

The reported symptoms differed largely between individuals with an anaphylactic reaction to wheat and individuals with self‐reported wheat sensitivity. The latter suffered mainly from gastrointestinal and other general symptoms such as malaise, fatigue, headache, joint and muscle pain. Such a symptom profile of self‐reported wheat sensitivity overlaps with the reported clinical symptoms of non‐celiac gluten sensitivity (NCGS), where leading gastrointestinal symptoms are abdominal pain, bloating and diarrhoea [28, 29, 30]. Other commonly reported extraintestinal symptoms were malaise, chronic fatigue, skin rash and headache [28, 29].

Cofactors can trigger allergic reactions and facilitate severe anaphylaxis [72, 73, 74, 75]. They have been reported promoting allergic reactions to various foods [7, 74, 76], but were mainly accounted for wheat‐induced allergic and anaphylactic reactions [26, 35, 77]. In particular, physical exercise and the intake of non‐steroidal anti‐inflammatory drugs (NSAIDs) were frequently described as cofactors in WEIDA [26, 35, 78, 79, 80]. Alcohol consumption was also observed as a further, less frequent cofactor in WDEIA [26, 34, 35]. Cofactors were also reported by patients in our study. Interestingly, individuals with self‐reported wheat sensitivity predominantly indicated psychological burden, alcohol intake and menstruation as possible cofactors (PAN‐WA), while physical exercise and medication intake were often reported in wheat anaphylaxis (NORA).

The majority of individuals (almost 87%) with self‐reported complaints due to food containing wheat made dietary adjustments in their daily lives. Up to a third of these individuals even strictly avoided wheat or gluten. Such an unnecessary avoidance behaviour can negatively affect not only the daily diet [81, 82] but also costs associated with dietary restrictions [81] and the quality of life of affected individuals [83].

## 5 Limitations

Our study focused on clinically confirmed wheat allergy and did not attempt to quantify NCGS. Although NCGS shares symptom overlap with self‐reported wheat sensitivity, its unclear pathophysiology and lack of validated biomarkers [84] prevent reliable epidemiological assessment. The willingness of the individuals contacted to participate in our study may have biased the results. The questionnaire survey achieved a response rate of only 12%, leading to the risk of an increased response rate among individuals interested in the topic of wheat allergy. This may have resulted in an overestimation of the calculated prevalence, which could possibly have been prevented by using a non‐response questionnaire. As more adults than legal guardians of adolescents completed the questionnaire, the data set for the age group of 12–17 years was limited. We were also unable to weight the data considering the educational level due to a lack of comparable data.

## 6 Conclusion

Besides differences in the prevalence between the perception of wheat sensitivity and the presence of a clinically proven wheat allergy, our data confirm the low frequency of wheat allergy in adults and adolescents in the general population. The distinguished symptoms (local gastrointestinal and non‐specific symptoms versus skin, respiratory and cardiovascular symptoms) easily allow a clinician to differentiate these entities in clinical practice. Our findings may help, for example, via web‐based communication, to improve knowledge of the low prevalence of wheat allergy and may support the avoidance of unnecessary dietary restrictions in a large proportion of the population.

## Author Contributions

A.N. contributed to the implementation of the research, performed the clinical study and the statistical analyses, and wrote the manuscript. S.D.B. contributed to the development of the study design, as well as to the implementation of the research, to the analysis of the results, and to the writing of the manuscript. V.H. participated in data collection, supported the statistical analyses, and reviewed and edited the manuscript. J.G. contributed to the development of the provocation scheme, supported the conduction of the clinical study, and reviewed and edited the manuscript. K.B. supported the implementation of the research and reviewed and edited the manuscript. M.W. supervised the study, contributed to the development of the study design, as well as to the implementation of the research, supervised the analyses of the results, critically reviewed the initial draft and assisted in writing the manuscript.

## Conflicts of Interest

The authors declare no conflicts of interest.

## Supporting information


Data S1.

## Data Availability

The data are not publicly available since they contain sensitive information that could compromise the privacy of participants. Data from this study can be requested from the corresponding author.
