# Supplementary material for: Prevalence and Clinical Symptoms of Wheat Allergy in Adults and Adolescents in Central Europe
Source: Clin Exp Allergy. 2025 Feb 19;55(4):319–29. doi: 10.1111/cea.70017 (PMC11994253; doi:10.1111/cea.70017)

**Supplementary Materials**

*Table S1 - Demographic data of all answered questionnaires (n=1,770).*

*^1^Weighting regarding the highest level of education was not possible due to a lack of comparable data.
^2^Equals the German educational level ´Hauptschulabschluss´.
^3^Equals the German educational level ´Realschulabschluss´.
^4^Equals the German educational level ´Fachschulreife´.
^5^Equals the German educational level ´Abitur´.
^6^Equals the German educational level ´Berufsausbildung´.
^7^Equals the German educational level ´Fachhochschulabschluss´.
^8^Equals the German educational level ´Hochschulabschluss´.*

*
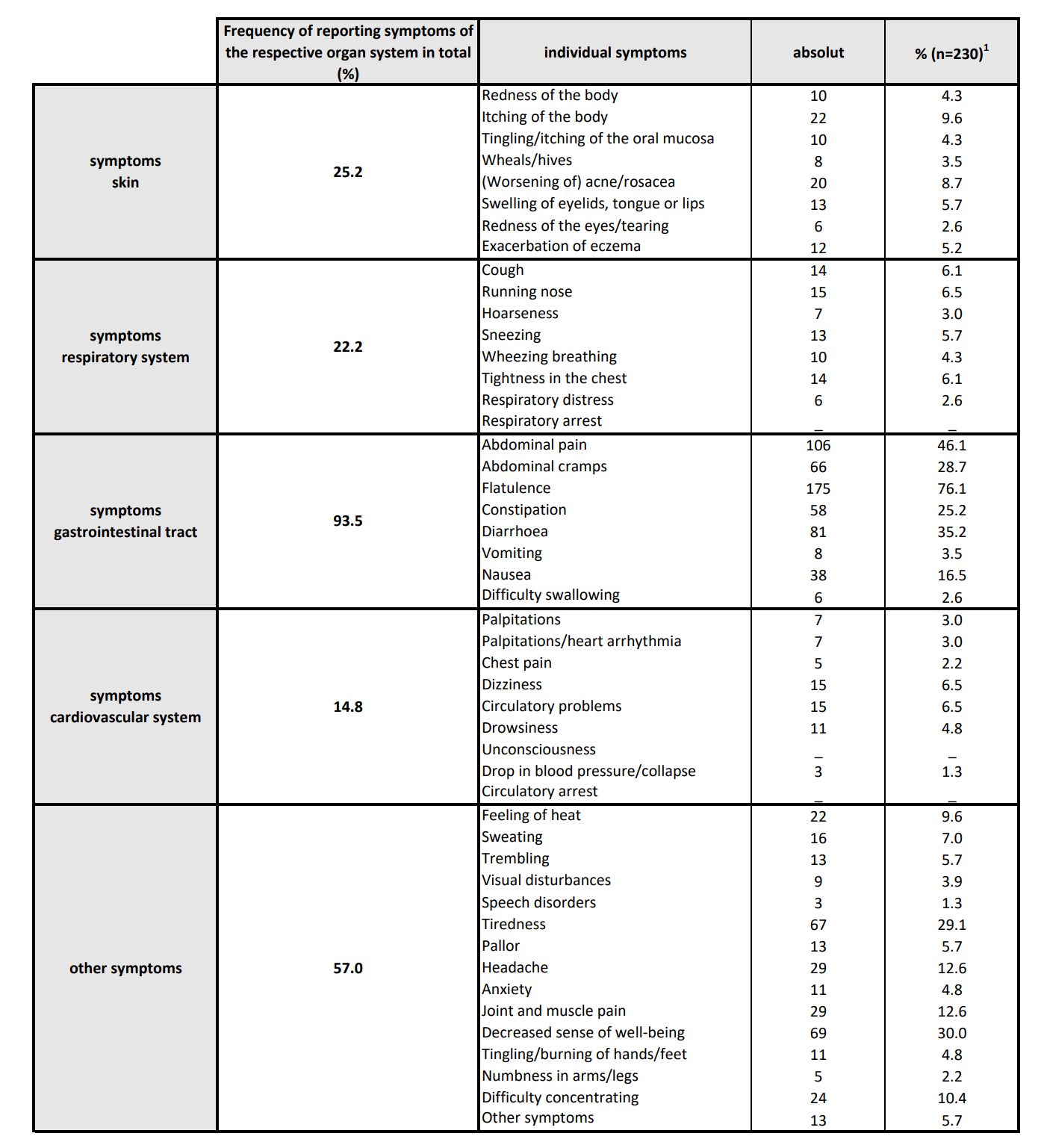
Table S2 - List of all symptoms stated in the questionnaire for self-reported wheat sensitivity, including the frequency of the affected organ systems.*

*^1^Number differs from n=232,* *as 2 individuals provided no answer.*

**Focused literature review**


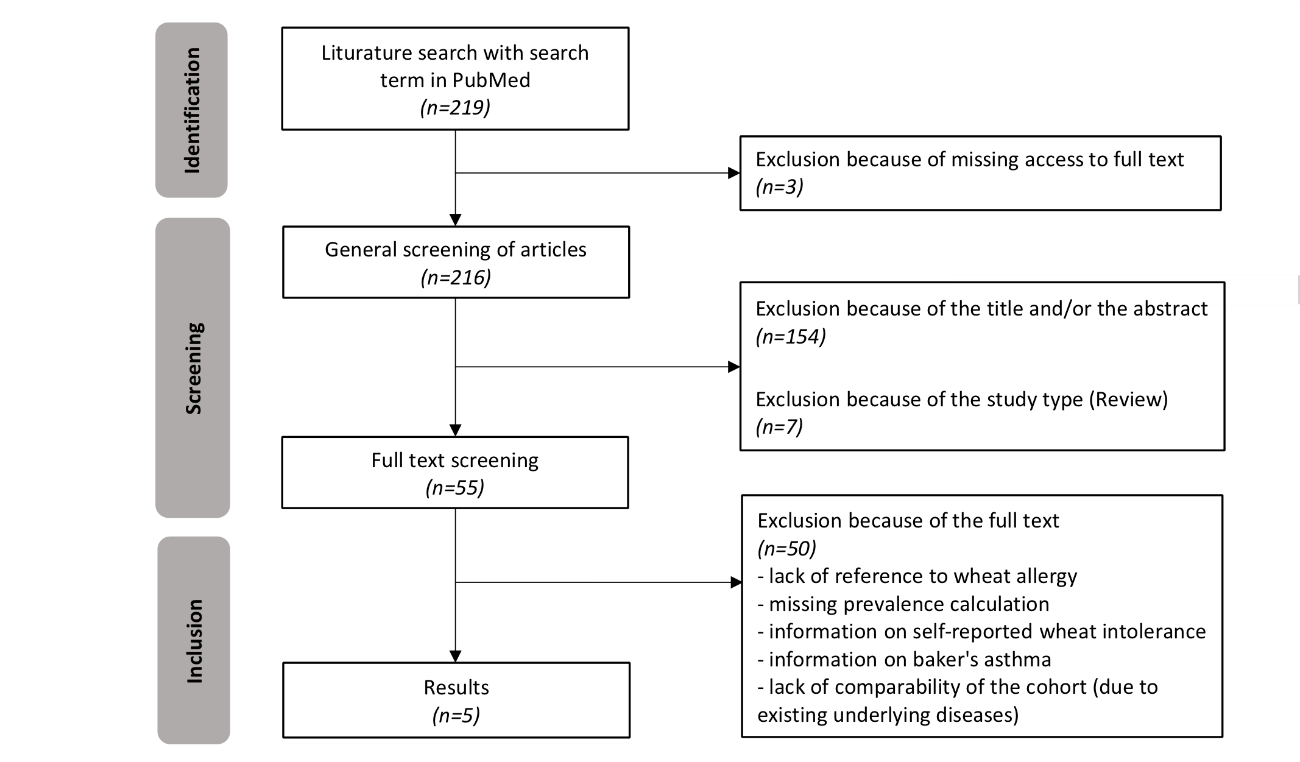
The focused literature review was conducted in the PubMed online database in November 2023. Five different categories were used for the search strategy based on Liu et al. (1): 1) “wheat” or “gluten” or “food”, 2) “allergy” or “hypersensitivity” or “anaphylaxis”, 3) “epidemiology” or “prevalence”, 4) “Europe” and 5) “cross-sectional study”. Only studies that were written in German or English and for which access to a full text was possible were included (via filter and subsequent control). It was also ensured that no duplicates were part of the analysis. Studies from all available years were evaluated (1987-2023). All study types that did not correspond to a cross-sectional study (e.g., reviews) were excluded. Figure S1 shows the process of the study selection.

*Figure S1 – Flowchart of the process of study selection.*

*Table S3 - Results from the focused literature review of the prevalence of wheat allergy in Europe.*

| **First author** | **Published year** | **Country** | **Sample** | **Prevalence** | **Diagnostic method** |
| --- | --- | --- | --- | --- | --- |
| Gaspar-Marques, J. (2) | 2014 | Portugal | Children (n=1,217) | 0.2% | Self-reported |
| Schmitz, R. (3) | 2013 | Germany | Children (n=12,988) | 9.9% | sIgE |
| Kallio, P. (4) | 2011 | Finland | Children (n=1,542) | 0.7% | Self-reported |
| Zuberbier, T. (5) | 2004 | Germany | All age groups (n=4,093) | 4.7% | Skin prick test |
| Pyrhönen, K. (6) | 2009 | Finland | Children (n=3,308) | 1.5%,  2.6% | Self-reported,  Self-reported physician-confirmed |

**References**

1. Liu W, Wu Y, Wang J, Wang Z, Gao J, Yuan J, et al. A Meta-Analysis of the Prevalence of Wheat Allergy Worldwide. Nutrients. 2023 Apr 1;15(7).

2. Gaspar-Marques J, Carreiro-Martins P, Papoila AL, Caires I, Pedro C, Araújo-Martins J, et al. Food allergy and anaphylaxis in infants and preschool-age children. Clin Pediatr (Phila). 2014 Jun 1;53(7):652–7.

3. Schmitz R, Ellert U, Kalcklösch M, Dahm S, Thamm M. Patterns of sensitization to inhalant and food allergens - Findings from the German health interview and examination survey for children and adolescents. Int Arch Allergy Immunol. 2013;162(3):263–70.

4. Kallio P, Salmivesi S, Kainulainen H, Paassilta M, Korppi M. Parent-reported food allergy requiring an avoidance diet in children starting elementary school. Acta Paediatrica, International Journal of Paediatrics. 2011 Oct;100(10):1350–3.

5. Zuberbier T, Edenharter G, Worm M, Ehlers I, Reimann S, Hantke T, et al. Prevalence of adverse reactions to food in Germany - A population study. Allergy: European Journal of Allergy and Clinical Immunology. 2004 Mar;59(3):338–45.

6. Pyrhönen K, Näyhä S, Kaila M, Hiltunen L, Läärä E. Occurrence of parent-reported food hypersensitivities and food allergies among children aged 1-4 yr. Pediatric Allergy and Immunology. 2009 Jun;20(4):328–38.

*Document S1 - Questionnaires of the PAN-WA study (one version for adults, one version for guardians of adolescents).*


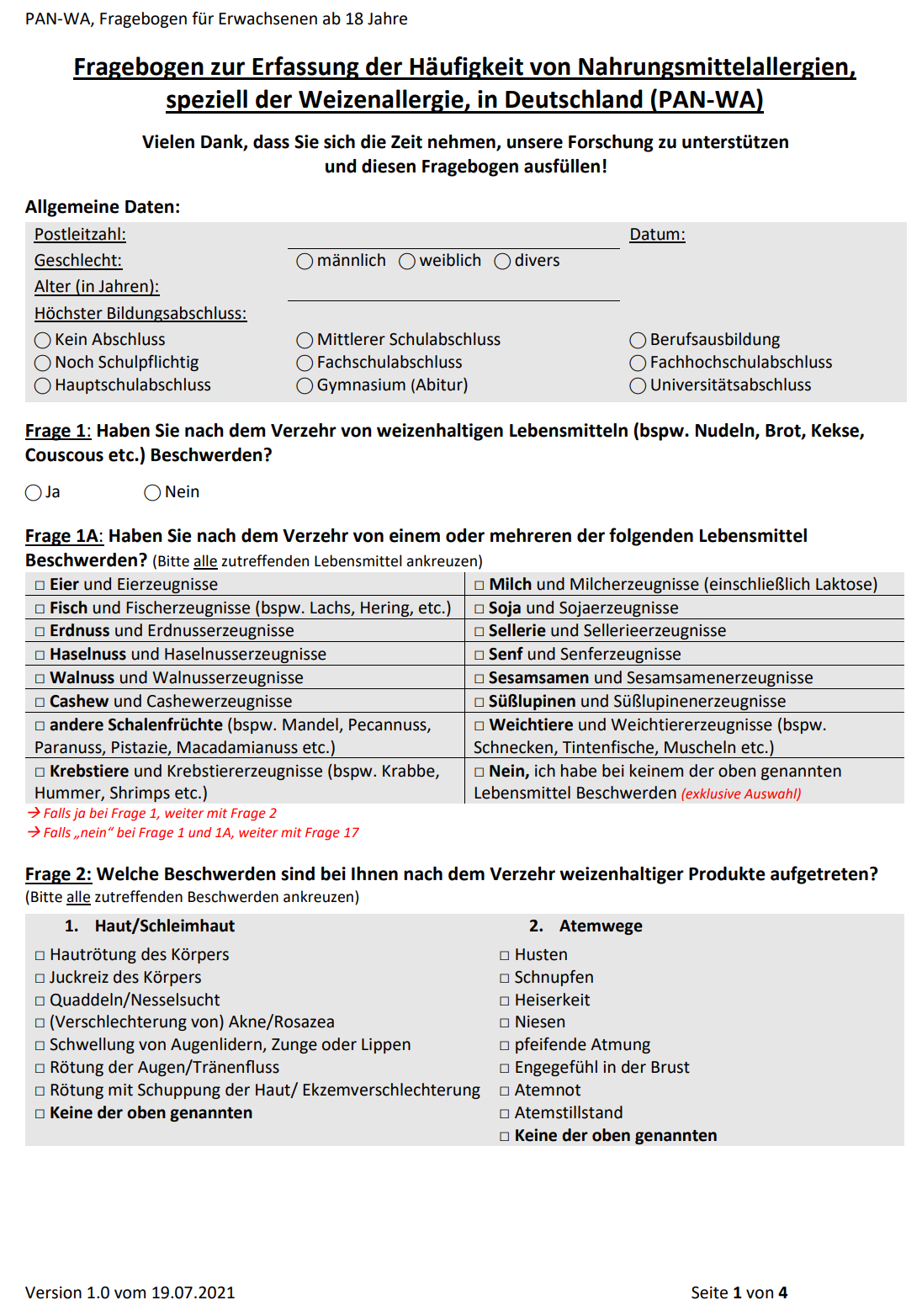


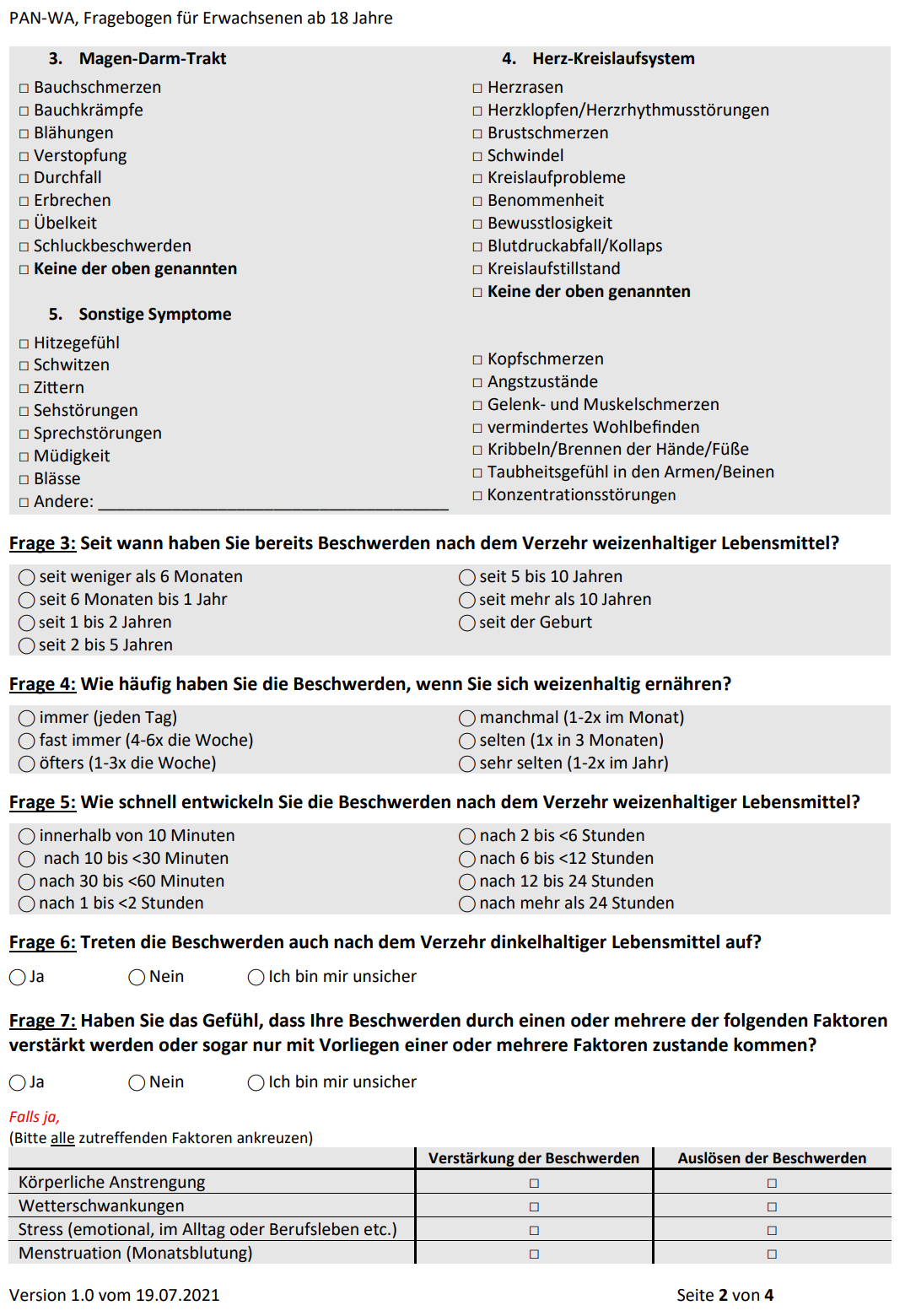


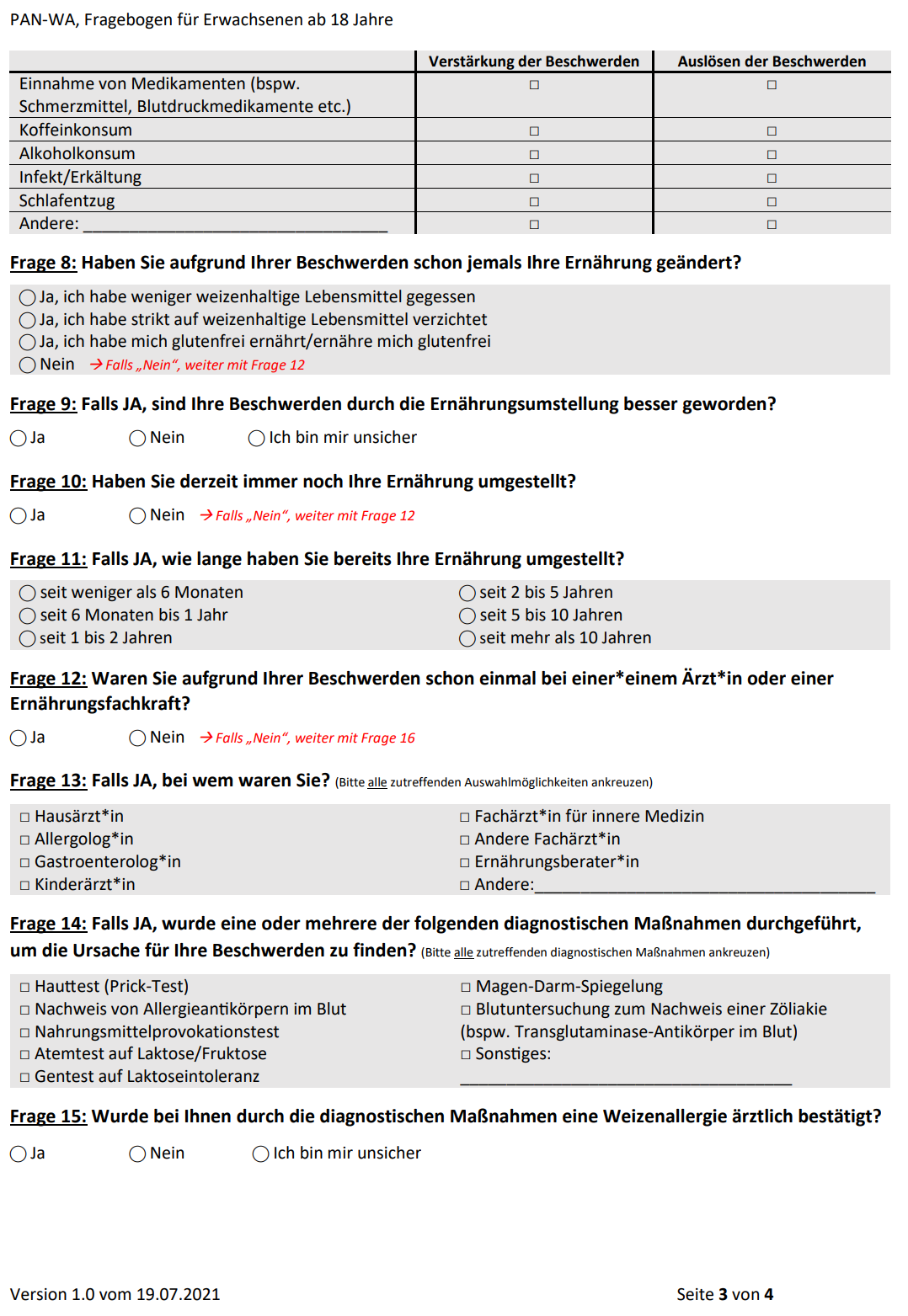


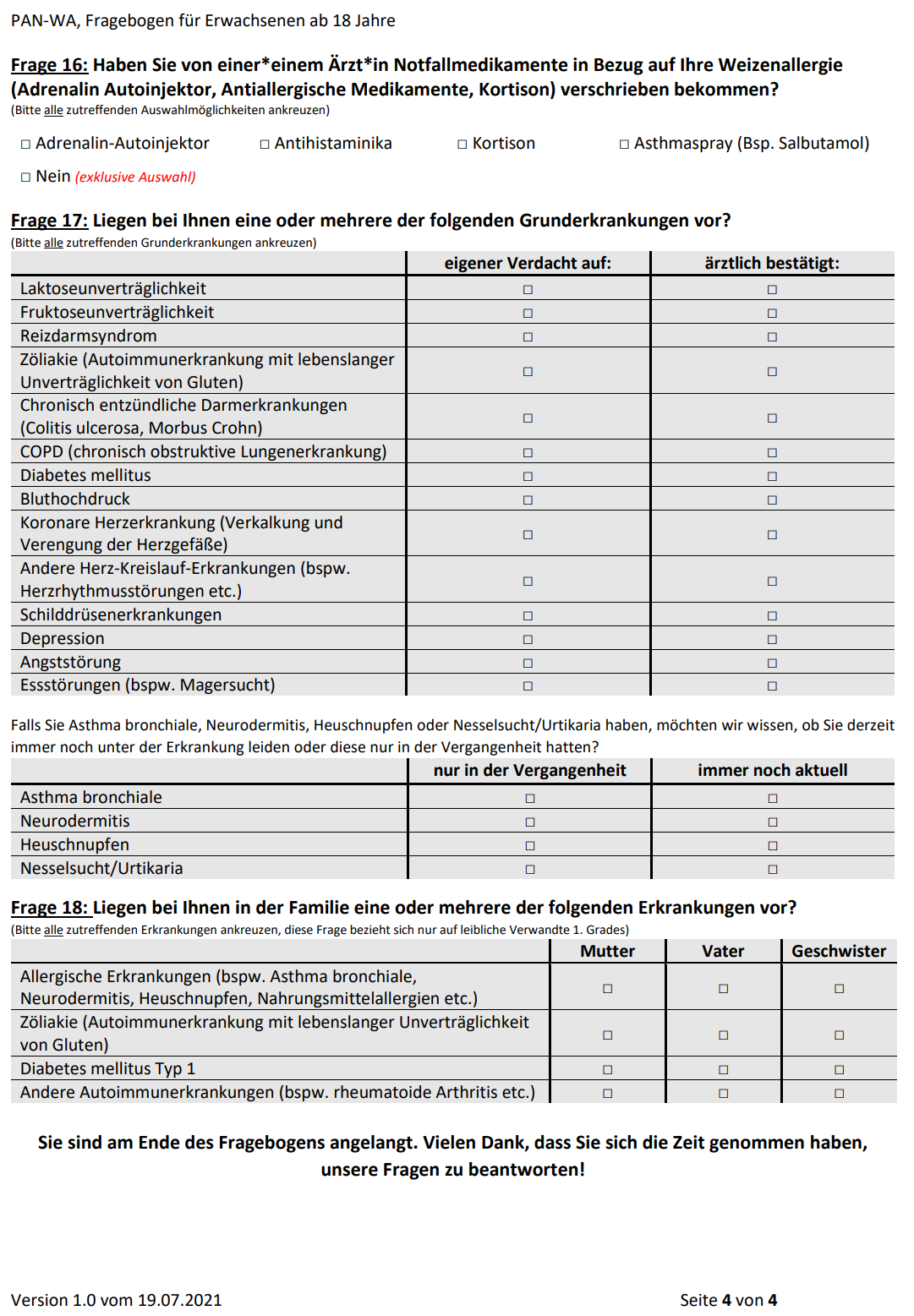


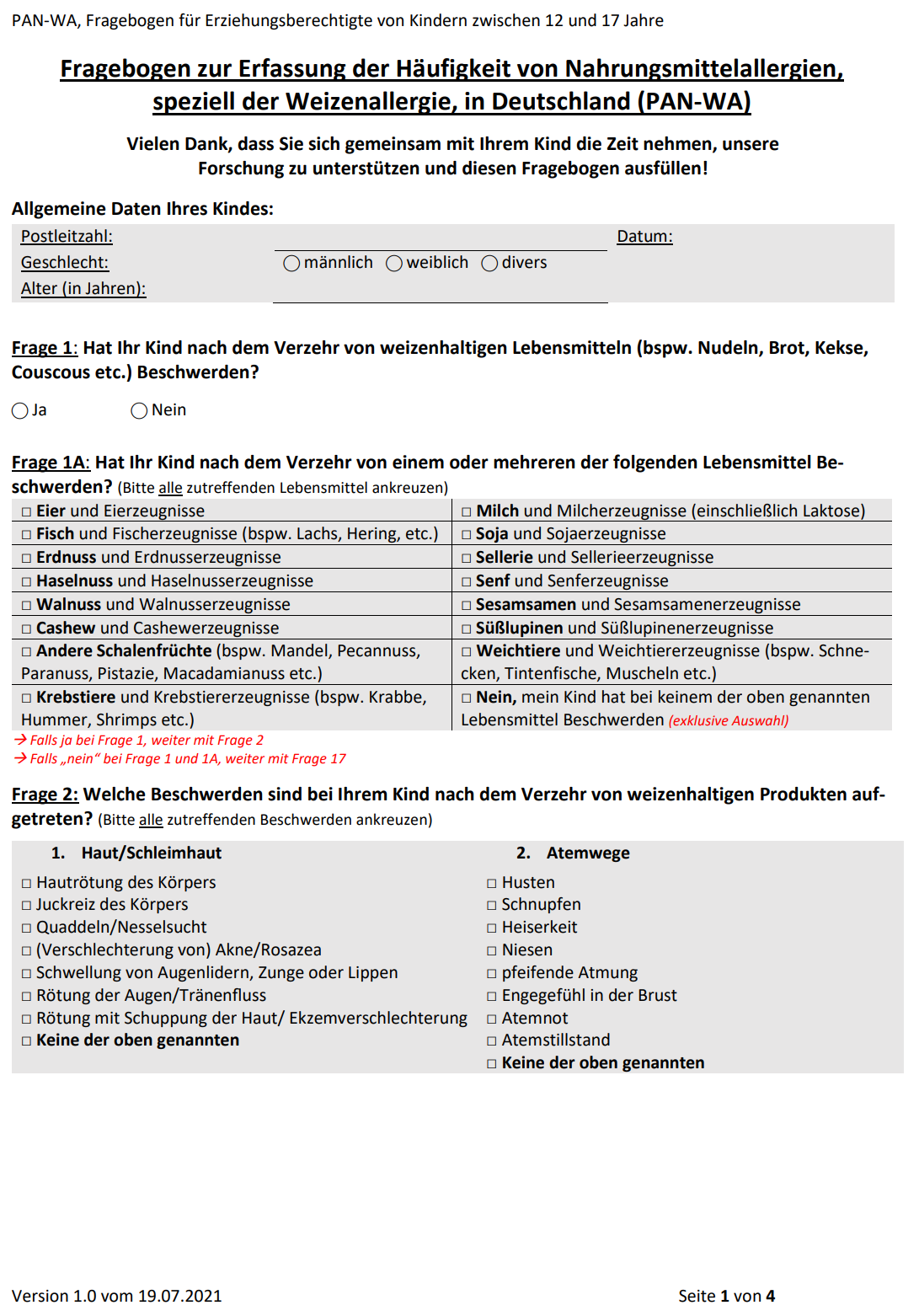


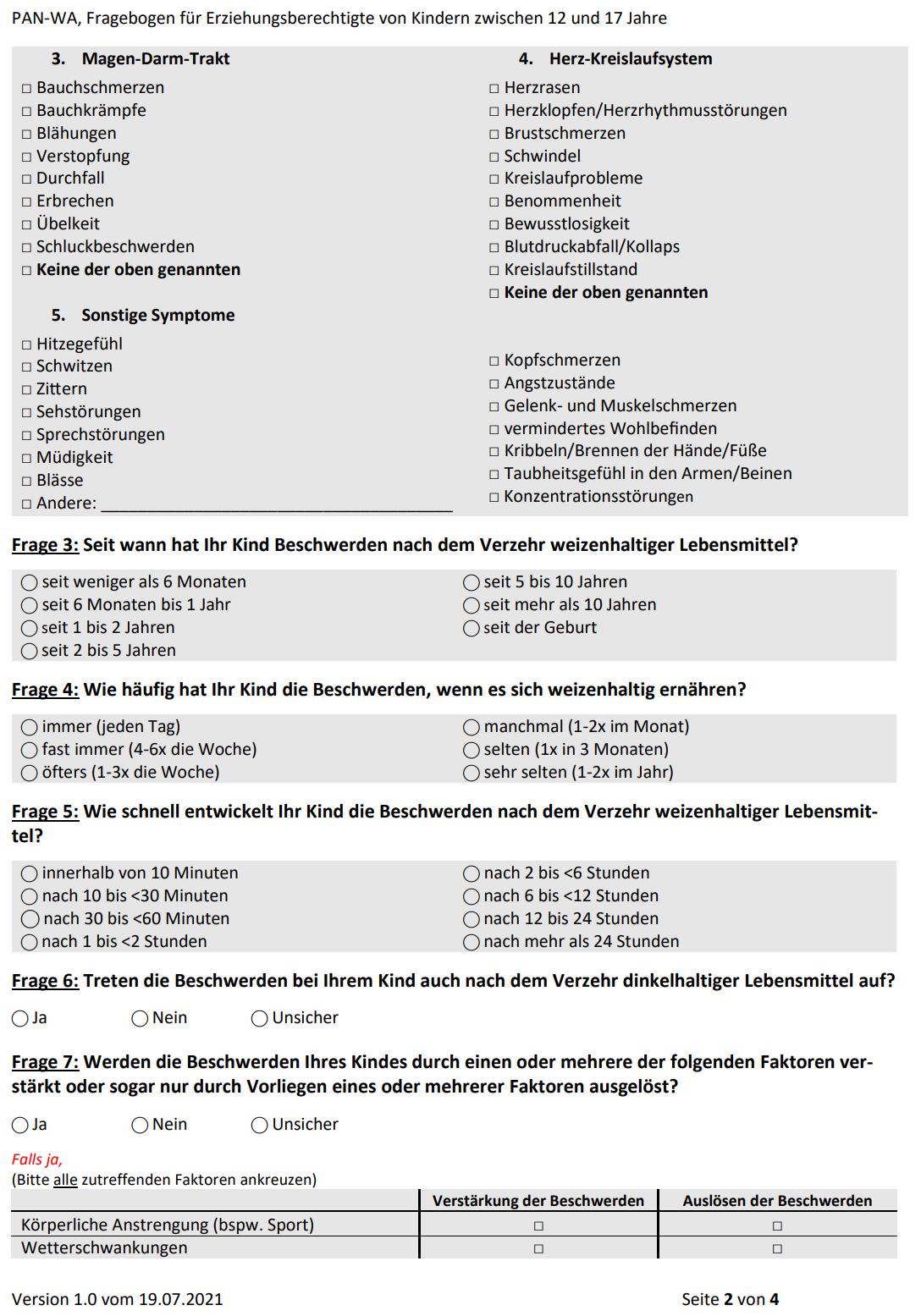


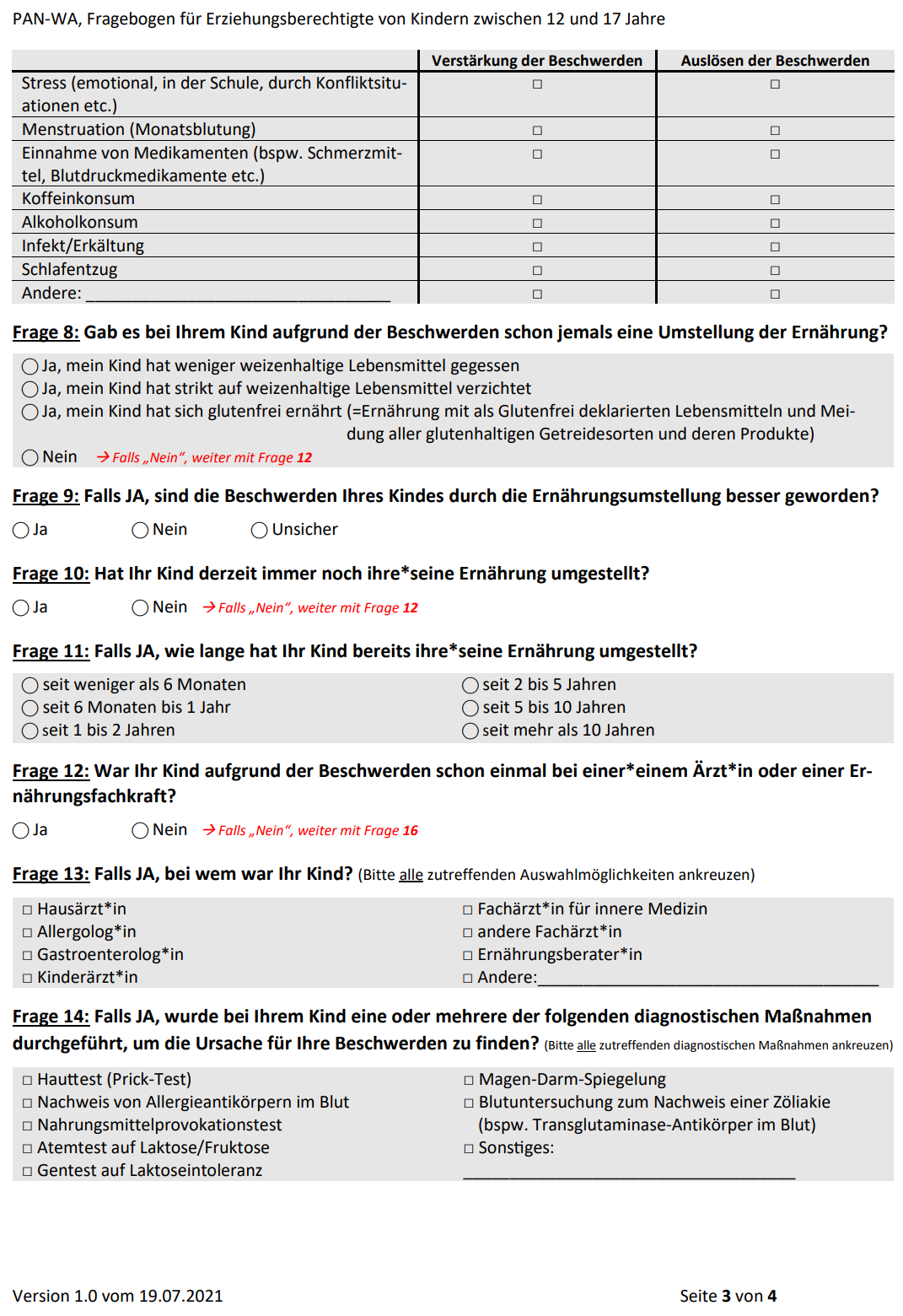


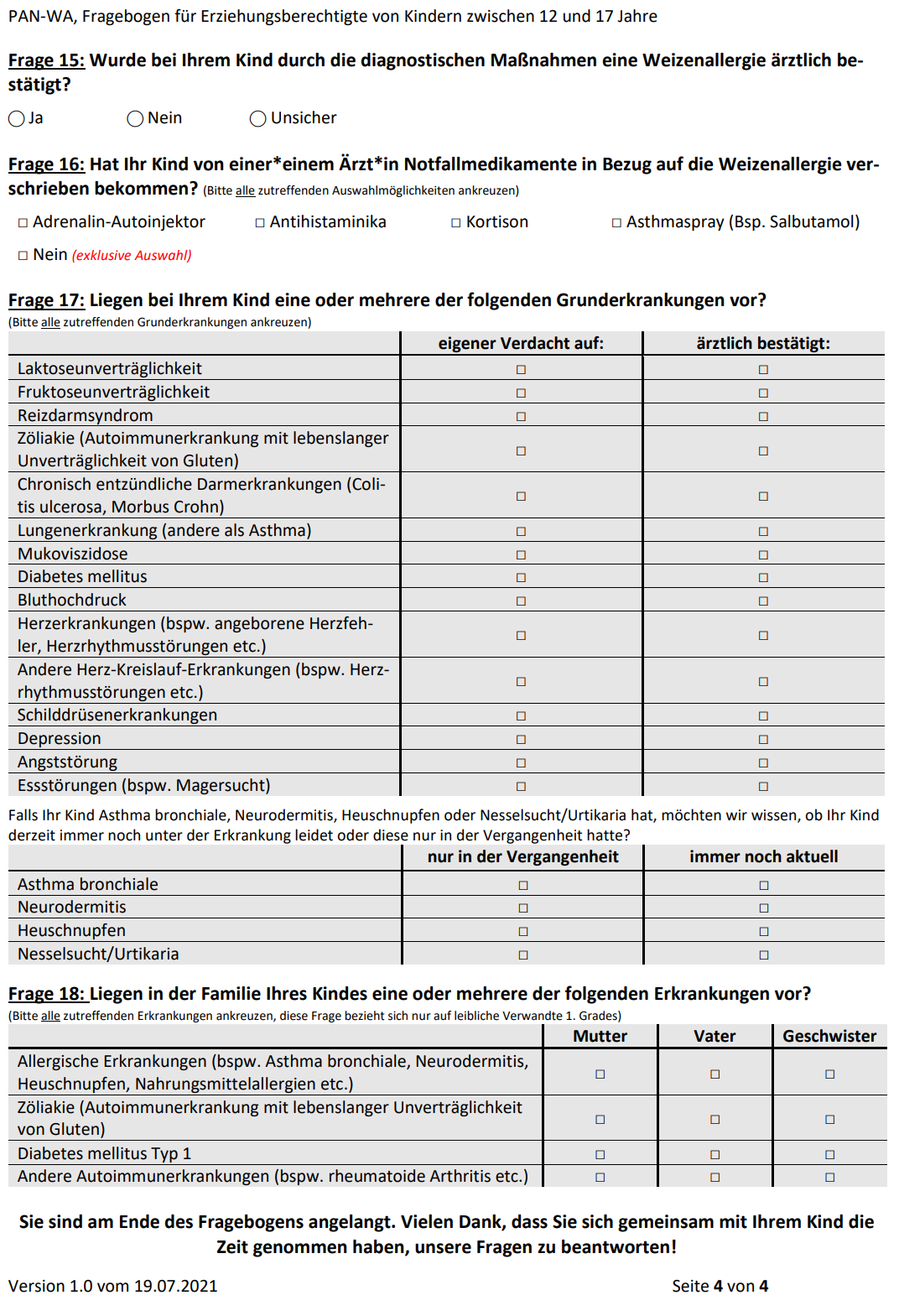

Supplement: Supplementary file 1 — Data S1. [file CEA-55-319-s001.docx]
